# Limits of experimental evidence in RNA secondary structure prediction

**DOI:** 10.3389/fbinf.2024.1346779

**Published:** 2024-02-22

**Authors:** Sarah von Löhneysen, Mario Mörl, Peter F. Stadler

**Affiliations:** ^1^ Bioinformatics Group, Department of Computer Science, Interdisciplinary Center for Bioinformatics, Leipzig University, Leipzig, Germany; ^2^ Institute for Biochemistry, Leipzig University, Leipzig, Germany; ^3^ Competence Center for Scalable Data Analytics and Artificial Intelligence, School of Embedded and Compositive Artificial Intelligence (SECAI), Leipzig University, Leipzig, Germany; ^4^ Department of Theoretical Chemistry, University of Vienna, Wien, Austria; ^5^ Facultad de Ciencias, Universidad National de Colombia, Bogotá, Colombia; ^6^ Center for Non-Coding RNA in Technology and Health, University of Copenhagen, Frederiksberg, Denmark; ^7^ Santa Fe Institute, Santa Fe, NM, United States

**Keywords:** RNA secondary structure, chemical probing, sequence covariation, pseudo-energies, loop-based energy model

## 1 Introduction

Chemical and enzymatic probing has a long history as an experimental source of information on RNA secondary structures. In recent years such protocols were interfaced with high-throughput sequencing methods to provide access to transcriptome-wide structural information (Kubote et al., 2015; Carlson et al., 2018). Despite the indisputable usefulness of structure probing, it is important to remember that any probing method provides a signal that *encodes* information on RNA structures, but remains far from directly measuring or unambiguously determining a structure.

An extensive body of empirical evidence of RNA structures has been integrated into the “standard model” for RNA secondary structure prediction. It defines an RNA secondary structure as a collection of Watson-Crick and GU base pairs such that i) each base has at most one pairing partner, ii) base pairs do not cross, i.e., if (*i*, *j*) is a pair, then there is no pair (*k*, *l*) with *i* < *k* < *j* and *l* < *i* or *l* > *j*, and iii) every base pair spans at least three unpaired positions (Lorenz et al., 2011). Every structure of this type is associated with an energy that can be computed as the sum of its loops (facets of its unique planar embedding), which correspond to stacked base pairs, hairpin loops, interior loops, and multi-branched loops. The energy contribution of each loop depends on its sequence, but is independent of its external context. Comprehensive tables of sequence-dependent loop energy contributions have been inferred (mostly) from melting experiments on small, specifically designed RNA molecules (Andronescu et al., 2014). Collected in the *standard energy model* (Turner and Mathews, 2010), they are used in exact dynamic programming algorithms that predict the ground state structure or the base pairing probabilities in the Boltzmann ensemble of secondary structures for arbitrary RNA sequences. We note in passing that Stochastic Context Free Grammars (SCFGs) use in essence the same model(s) for the structures (Rivas et al., 2012) and may serve as an alternative to the thermodynamic approach. Usually, SCFGs are parametrized using learning approaches from known structures, see, e.g., (Do et al., 2006). For the purpose of the present contribution, it is only important that there is a “universal” model that predicts (a reasonable approximation of) the secondary structure taking an arbitrary RNA sequence as input.

Empirical evidence, e.g., from probing experiments can be included in the universal structure prediction methods as a hard constraint forbidding structures that contradict the empirical evidence or as an additional energy term (soft constraint), favoring those structures that conform better to the empirical data over others, see, e.g., (Lorenz et al., 2016c; Lorenz et al., 2016a). Since *unambiguous* experimental evidence that a base is paired or unpaired is difficult to obtain, we consider here only soft constraints that better reflect the probabilistic nature of the available evidence. This amounts to the inclusions of pseudo-energy terms that award a “bonus” to all secondary structures that exhibit a specific feature whose presence is supported by the external evidence.

## 2 Pseudo-energies from probing data

Most current methods for large-scale chemical probing use deep sequencing methods as read-out. The raw signal thus is a number of reads associated with each sequence position *i*. The probing methods most frequently employed at present detect unpaired positions. In SHAPE, RNA forms an adduct at conformationally flexible 2′-hydroxyl positions (Deigan et al., 2009), where flexibility serves as a proxy for unpairedness. Similarly, DMS treatment leads to a methylation of N^1^ of adenine and N^3^ of cytosine in unpaired bases. In inline probing and related protocols using heavy metal ions as catalysts, the RNA is cleaved preferentially at unpaired positions. Both cleavage and bulky adducts (which lead to termination of reverse transcription) translate to read-ends in subsequent high-throughput sequencing.

Other chemically introduced modifications, in particular at the 2′-hydroxyl position of the ribose of structurally unconstrained positions, lead to misincorporations during cDNA synthesis, because reverse transcriptases incorporate non-templated nucleotides (Smola and Weeks, 2018). In the SHAPE-MaP approach, the nature of the misincorporated base can be identified as a base replacement in the resulting sequence alignments (mutational profiling). However, the efficiency of the ribose modification depends on the reactivity of the 2′-hydroxyl group, which itself is affected by the nature of the individual base (Wilkinson et al., 2009; Busan et al., 2019). Hence, the number of reads that is used as a proxy for signal reliability can vary to a certain extent. PORE-cupine, the combination of such structure-dependent nucleotide modifications with the Nanopore sequencing technology allows for an elegant direct read-out of these signals in individual RNA molecules, enabling single molecule structure analysis (Aw et al., 2021).

High throughput probing experiments require two distinct normalization steps since the observed, position-wise signal depends i) on the abundance of the probed RNA, i.e., the expression level, and ii) on the secondary structure. Normalization for expression levels requires annotated transcripts. While it would be desirable in principle to have RNA-seq data for the same sample to estimate expression levels, and possibly to refine the annotation, it is possible to use the probing signal itself. A reasonable normalized signal *S* can be obtained, e.g., by dividing the counts by the mean (adjusted to drop outliers) or the median of the read counts over a given annotation item. Much more elaborate statistical models have been developed to estimate *reactivities* from high-throughput sequencing data, taking into account both RT stops and misincorporations (Strobel et al., 2018; Yu et al., 2018).

The normalized signal then needs to be related to a probability or pseudo-energy contribution for the feature under consideration. Reactivities are often directly converted to pseudo-energies using simple empirical formulas (Strobel et al., 2018). A more principled approach proposed by Zarringhalam et al. (2012) is to first estimate the probability *p*(*S*) of the features as a function of the observed signal strength and then to convert *p*(*S*) to a pseudo-energy via

$$E\left(S\right)=-RT\ln\frac{p\left(S\right)}{1-p\left(S\right)}$$ (1)

Here *p*(*S*) will in general be a monotonic function of the normalized signal. Typically, *p*(*S*) will be sigmoidal to limit the impact of outliers. We note in passing that the conventional conversion of SHAPE reactivities to pseudo-energies (Low and Weeks, 2010), *E* = *m* ln(*S* + 1) + *b*, is a particular case of Eq. 1 using the sigmoidal function *p*(*S*) = 1/[1 + (*b*/*RT*)(*S* + 1)^*m*/*RT*^] with empirical fitting parameters *b* and *m*. The pseudo-energy terms are included as additional position-dependent contributions in the thermodynamic RNA folding algorithms, see e.g., (Lorenz et al., 2016c; Lorenz et al., 2016b).

The advantage of Eq. 1 is not only a more direct interpretation as a log-odds ratio. It is also readily extended to aggregating evidence from different sources, e.g., from different probing experiments. This is used in practice, e.g., in the Led-Seq approach (Kolberg et al., 2023). There, each lead-induced cleavage at single-stranded positions is assayed both via the 2′,3′-cyclophosphate end and the 5′-OH end and modeled via a two-dimensional sigmoidal fit *p*(*S*_1_, *S*_2_). Of course, it is also possible to stratify the signal for instance by the identity of the cleaved di-nucleotide. The function *p*(*S*) can be estimated by comparing the observed signal *S* with the frequency of the assayed feature in a set of reference structures. Typically, a collection of well-known structures is used for this purpose. It should be kept in mind, however, that few RNA structures are perfectly known at present. Recent advance in Cryo-EM methods, however, may alleviate this bottleneck (Ma et al., 2022). Moreover, RNA structures can change with temperature (Narberhaus et al., 2006; Marz et al., 2010), salt concentration (Yao et al., 2023) and with the presence of binding partners such as proteins or other RNAs (Sutandy et al., 2018). In practice, therefore, the calibration of the function *p*(*S*) will have to be performed from imperfect data.

**FIGURE 1:**
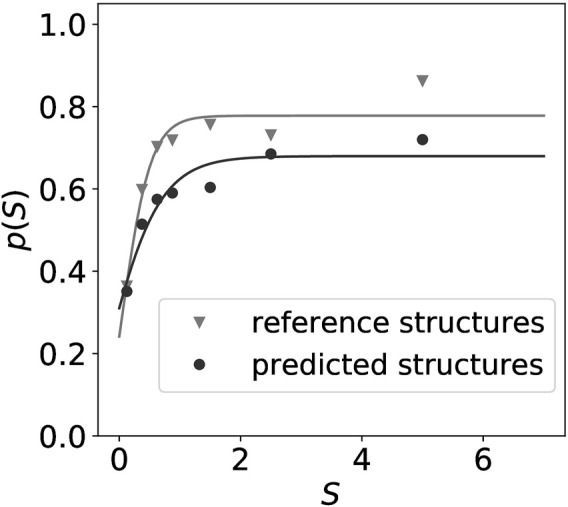
Normalized intensities *S* obtained from probing experiments [in this example from a Led-Seq cP library (Kolberg et al., 2023)] can be converted to probabilities of a structural feature (here the probability of a sequence position to be unpaired) by relating the empirical signal in a bin [*S*, *S* + Δ*S*] to the frequency of the feature of interest (here the frequency of observing an unpaired position) in a reference set. Here we compare a manually curated set of 32 reference secondary structures from *Escherichia coli* (▿) to secondary structures predicted from the thermodynamic model using the ViennaRNA software (•) of all sequences with valid probing information from the same study (Kolberg et al., 2023). A smooth function *p*(*S*) is then obtained by fitting a sigmoidal curve to the empirical data.

Figure 1 shows that the thermodynamic model for RNA folding is surprisingly accurate in predicting the distinction of paired versus unpaired nucleotides. Predicted structures can therefore serve as an alternative to reference structures when estimating *p*(*S*). The difference in accuracy between a manually curated reference set and predictions from the standard energy model translates to a lower saturation value in terms of *p*(*S*) for the thermodynamic predictions. Importantly, the curve for the curated reference data also saturates at a value well below the theoretical upper bound of *p*(*S*) = 1 for *S* → *∞*. While possible errors in the reference data may contribute to this effect, it is most likely dominated by the fact that probing methods assay chemical properties that are only correlated with structural features such as the unpairedness of a nucleotide instead of directly measuring them.

Moreover, several RNA molecules are known to contain high affinity binding sites for metal ions (Pyle, 2002) that can lead to disproportionately high cleavage results (Ciesiolka et al., 1994). Nevertheless, such prominent cleavage sites are highly informative in structural analysis based on metal cleavage (Behlen et al., 1990; Kolberg et al., 2023). Another point to consider is the fact that temperature can have a dramatic impact on the probing result. Here, lead-dependent probing can be applied to a wide range of temperatures, allowing for structural investigation in psychro-as well as thermophilic organisms (Kolberg et al., 2023). Other approaches depending on more temperature-sensitive reagents might be limited in this aspect, and the corresponding half-lives at increased temperatures must be considered (Smola and Weeks, 2018; Busan et al., 2019).

The distribution of errors in thermodynamic secondary structure predictions could in principle be determined empirically by comparison with curated reference data. This opens the possibility to devise estimates for *p*(*S*) that compensate for the imperfections of thermodynamic predictions. We are not aware, however, that such a method has become available.

## 3 Pseudo-energies from phylogenetic conservation

Evolutionary conservation of structures leads to mutual constraints at spatial contacts. Given a multiple sequence alignment, this effect can be quantified as covariation or mutual information between alignment columns. In MIfold (Freyhult et al., 2005), consensus secondary structures are predicted with reasonable accuracy directly from the mutual information of alignment columns. The thermodynamic model can also be readily extended to multiple sequence alignments by averaging energy contributions for stacking and loops over the rows (Hofacker et al., 2002; Bernhart et al., 2008). In practice, the RNAalifold program also uses covariance-based pseudo-energies. Both MIfold and RNAalifold are based on the assumption that there is a global consensus structure that is present in each of the aligned sequences in essence without variations. This is not always the case, however.

In many cases evolutionary conserved secondary structures are only local elements in often much larger RNA molecules. This is most obvious for features such as Selenocystein Insertion (SECIS) elements or structured Internal Ribosomoal Entry Sites (IRES) on protein-coding mRNAs. Conserved structures in long non-coding RNAs also seem to be local in general. In such cases, consensus methods typically predict large unstructured regions. For any particular RNA molecule, these regions will typically form secondary structures, which, however, are not conserved across the aligned sequences. von Löhneysen et al. (2023) therefore proposed to convert predicted consensus structures into pseudo-energies. In the setting of the RNAalifold approach this is most directly achieved by using $\Gamma_{ij}=\min\left\{0,-RT\ln\left[{p{}^{\circ}}_{ij}/\left(1-{p{}^{\circ}}_{ij}\right)\right]\right\}$ as pseudo-energy, where *p*°_*ij*_ is the probability of *i* and *j* being paired in the alignment according to the averaged energy model. The values of Γ_*ij*_ are truncated at 0 since the absence of conserved base pairs does not imply that the two positions are excluded from base pairing in particular sequences. The statistical significance of covarying alignment columns can be tested using Rscape (Rivas et al., 2017).

Not surprisingly, the beneficial effect of conservation-derived pseudo-energies on the accuracy of the structure prediction depends strongly on the quality of the multiple sequence alignment (von Löhneysen et al., 2023). In addition, the phylogenetic distribution of the input sequences may play a role. Some consensus structure prediction methods such as pfold (Knudsen and Hein, 2003) explicitly require a phylogenetic tree. Alternatively, the sequences (rows) in an alignment may be given weights depending on the similarity to the other aligned sequences (Vingron and Sibbald, 1993); this is used, e.g., in RNAalifold (Bernhart et al., 2008). Both explicit and implicit phylogenetic information incurs the danger, however, that the effect of alignment errors are aggravated. Misaligned sequences can be expected to have larger distances from other members of the alignments. As a consequence they appear less redundant and thus contribute with higher weight.

## 4 External evidence versus energy-based prediction

**FIGURE 2:**
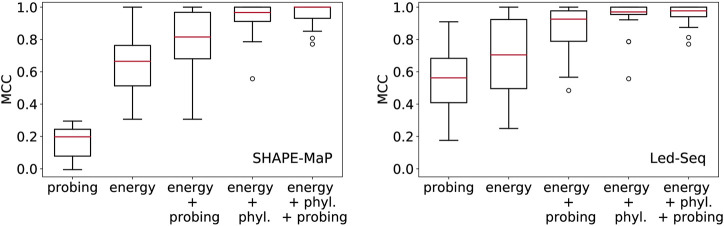
Structural information in probing data and comparative analysis of RNA structures. Position-wise probing information alone yield less accurate structures. Combining probing or conservation data (phyl.) with the energy model improves predictions considerably. Left: SHAPE-Map data of 12 ncRNAs from *Escherichia coli* (Mustoe et al., 2018) downloaded from RASP (Li et al., 2020), Right: Led-Seq data of 24 ncRNAs from *Escherichia coli* (Kolberg et al., 2023). Probing data were converted to pseudo-energies as described by Kolberg et al. (2023) for probing data and by von Löhneysen et al. (2023) for phylogenetic information. MCC, Matthews correlation coefficient.

In Figure 2 we compare the accuracy of RNA secondary structures generated by using different combinations of evidence. For the sake of this exposition, we only analyzed a limited data set. While this cannot replace a thorough benchmarking, it shows the salient trends, and illustrates some basic facts.

In order to assess the structural information contained in the probing signals alone, we exclusively used the position-wise pseudo-energies as energy model. The contribution for potential base pair (*i*, *j*) is set to *E*_*ij*_ = −(*E*_*i*_ + *E*_*j*_), where *E*_*i*_ and *E*_*j*_ is the pseudo-energy for positions *i* and *j* to be unpaired. Since this “energy model” scores the base pairs and unpaired positions independently of each other, the optimal secondary structure can be computed using Nussinov’s circular matching algorithm (Nussinov and Jacobson, 1980) in the same manner as for the calculation of maximum expected accuracy structures (Lu et al., 2009). For both SHAPE-MaP and Led-Seq data, the predicted structures are rather inaccurate. In particular, the probing data alone yield structures that are systematically worse than the un-aided energy-based predictions. We note that the SHAPE-MaP and Led-Seq data in Figure 2 are not directly comparable since they were retrieved from unrelated experiments. In SHAPE-MaP, Led-Seq, and DMS probing, structural signals are only obtained for unpaired nucleotides, while base-paired regions are inert. The protocol commonly employed for SHAPE data is to interpret low signals as a double-stranded segment in the RNA structure. Here, the misinterpretation of unreacted but single-stranded regions is possible, resulting in misleading structural models. Therefore Kolberg et al. (2023), instead interprets strong signals as unpaired regions, reducing the danger of misreading the lack of signal.

Including either probing data or conservation information substantially improves the structure prediction. This shows that the experimental evidence from probing can be meaningfully accessed only in conjunction with a universal folding model, in our example the thermodynamic model. We also observe that the inclusion of phylogenetic information yields substantially better structural models than the probing data. This is probably a consequence of the fact that probing data offer only position specific constraints, while phylogenetic methods introduce specific base pairs and thus restrict the search space quite drastically.

## 5 Discussion

The main purpose of this short opinion piece is to highlight the fundamental importance of the loop-based thermodynamic energy model (or one of its SCFG-based variants) for RNA secondary structure determination. Although chemical and enzymatic probing methods provide invaluable additional structural information, they cannot unambiguously determine RNA structures on their own. This begs the question to what extent probing data can identify pseudoknotted structures and whether this can be achieved without a reasonably accurate pseudoknot-aware thermodynamic folding algorithm.

Tb-seq exploits the fact that Tb^3+^ causes backbone cleavage in regions where the compression of the phosphate backbone causes sharp, stable turns in the RNA structure. Since such regions are typically associated with stable tertiary interactions, Tb-seq provides key information beyond secondary structures (Patel et al., 2023), even though its signal yields a position-dependent profile just like other probing methods. The interpretation of Tb-seq data at present also requires a good structural model to start with.

The information obtainable from crosslinking approaches such as SPLASH, RIC-seq, PARIS, LIGR-seq and others [reviewed, e.g., by Zhang et al. (2022)] goes beyond position-wise base pairing propensities and provides direct evidence on interacting RNA regions, see, e.g., (Schäfer and Voß, 2021). It seems fair to say, however, that the problem of deriving detailed secondary structure models from such data has not yet been solved in a satisfactory manner. Comparative sequence analysis provides an attractive alternative since it yields direct evidence on specific base pairs. This information can then be included into folding algorithms in the same way as probing data, namely, be adding a pseudo-energy *E*_*ij*_ to the base pair (*i*, *j*). Wherever comparative data on a conserved structural consensus is applicable and available, furthermore, these tend to have a larger beneficial impact on prediction accuracy than probing data. We believe that this is due to the fact that the specific consensus base pairs are much more informative than the position-wise status of being paired or unpaired. It appears, furthermore, that very little is gained by combining conservation and probing information in cases where the entire RNA structure is well-conserved over a long evolutionary time scale. On the other hand, probing data are invaluable in the much more frequent scenario that only certain functional elements of an RNA are well-conserved. In this setting we expect that the combination of probing and conservation data is particularly useful.

The energy-directed model also seems to be sufficient at least in principle to gauge the conversion of (normalized) probing signals to pseudo-energies. This alleviates the need to build large collections of manually curated reference structures, which would be hard to obtain in many cases, in particular when analyzing transcriptomes of non-model organism. Less naïve methods than the simple fitting procedure of Figure 1, however, will need to be developed for this purpose.

Despite these limitations of probing data and the superiority of comparative information, where it is available, probing is indispensable in many situations. For example, structural changes caused by binding partners or chemical modifications are difficult, and usually impossible, to capture by conservation data, even though it is possible in some cases to identify conserved alternative folds, see, e.g., (Meyer, 2017). Similarly, probing data are key whenever phylogenetic evidence does not exist, as is the case for RNAs designed in synthetic biology applications (Domin et al., 2017) and in evolutionary novelties appearing in loci with accelerated evolution (Beniaminov et al., 2008).
